# *Allolobophora caliginosa* coelomic fluid and extract alleviate glucocorticoid-induced osteoporosis in mice by suppressing oxidative stress and regulating osteoblastic/osteoclastic-related markers

**DOI:** 10.1038/s41598-023-29070-5

**Published:** 2023-02-06

**Authors:** Marwa Ahmed Abdelfattah, Ayman Saber Mohamed, Sherif Abdelaziz Ibrahim, Sohair R. Fahmy

**Affiliations:** Zoology Department, Faculty of Science, Cairo University, Giza, 12613 Egypt

**Keywords:** Molecular biology, Physiology, Diseases

## Abstract

*Allolobophora calignosa* (Ac) is a folk medicine for millennia, as it possesses many biological activities. This study aimed to investigate the chemo-preventive activity of *A.calignosa* coelomic fluid (AcCF) and *A.calignosa* extract (AcE) on glucocorticoid-induced osteoporosis (GIOP) in mice. Characterization and in vitro biological activity of AcE and AcCF has been assessed. Male CD-1 mice were subcutaneously received dexamethasone (DEX) (1 mg/kg, 5 times/week) and concurrently intraperitoneally treated with either AcCF (20 mg/kg) or AcE (45 mg/kg) every other day for 28 days. Serum and bone homogenates were subjected for qPCR and biochemical analysis. AcE and AcCF treatment significantly increased bone mineral density (BMD), bone mineral content (BMC), calcium (Ca), phosphorus (P), and calcitonin levels, whereas activity of serum alkaline phosphatase (ALP), bone alkaline phosphatase (BALP), serum acidic phosphatase (ACP), bone acidic phosphatase (BACP) and parathyroid hormone (PTH) levels were significantly reduced compare with untreated GIOP mice. Treatment with AcE and AcCF modulates oxidative stress and downregulated *Rank* and *Mmp9* expression, as well as increased glycosaminoglycan content in the organic bone matrix, resulting in osteoclastogenesis inhibition. Overall, AcCF and AcE show a chemo-preventive activity against GIOP by inhibiting oxidative stress and regulating expression and/or activity of osteoblast/osteoclast-related markers.

## Introduction

Glucocorticoids (GCs) are widely utilized to treat various systemic disorders and are important anti-inflammatory and immunosuppressive agents^1^. In contrast, long-term use of GCs promotes bone loss by destroying osteocytes and osteoblasts and increasing reactive species generation^2^. The synthetic long-acting GC, dexamethasone (DEX), causes disturbance in bone homeostasis^3^. Three types of cells maintain bone homeostasis: osteoblast, osteocyte, and osteoclasts; Osteoclast is responsible for breaking down the extracellular matrix; osteoblast and osteocyte are accountable for building the extracellular matrix up, known as osteoid. Thus two processes undergo a continuous cycle of bone modelling and remodelling^4^. The receptor activator of nuclear factor-kappa B (NF-κB) ligand (RANKL) is a membrane-associated cytokine produced by osteoblasts^5^. When RANKL binds to the receptor RANK, made from osteoclast precursor, the remodelling process is activated, leading to osteoclastogenesis and the breakdown of the extracellular matrix^6^. Osteoprotegerin (OPG) is a soluble decoy receptor for RANKL made by osteoblast. OPG rule inhibits the RANK/RANKL interaction, inhibiting osteoclast overproduction even though elevated osteoclast activity culminates in bone resorption rather than bone remodelling^7^.

Furthermore, GCs can indirectly affect bone by lowering gastrointestinal calcium absorption and increasing renal calcium loss; altering calcium homeostasis leads to hyperparathyroidism, which increases osteoclast activity^8^; thus, the most prevalent type of secondary osteoporosis is glucocorticoid-induced osteoporosis (GIOP). Patients who take glucocorticoids must also take anti-osteoporosis medicines to prevent the onset and progression of GIOP^9^.

In treating osteoporosis, natural products have been demonstrated to be very effective^10^. Because of the medicinal importance of earthworm, it has been adopted as a natural treatment for many diseases^11^. It has a high nutritional value due to its soil origin^12^. Thus, it has been consumed in various ways; in previous years, earthworm was advised to be edible for anemia and malaria^13^. Recent research dedicated that *Allolobophora calignosa,* which is an earthworm species that belong to the phylum Annelida^14^, possesses many activities such as antioxidant^11^, anticoagulant, antibacterial and anticancer^15^.

Because inflammation and oxidative stress are intricately connected to osteoporosis progression, *Allolobophora calignosa* coelomic fluid (AcCF) and *Allolobophora calignosa* extract (AcE) are hypothesized to have antiosteoporosis capabilities^16^. So, this study aimed to investigate the possible anti-osteoporotic effect of AcE and AcCF against GIOP in male mice.

## Materials and methods

### Extraction of AcE and AcCF

For the study, earthworms (about 1 kg) were purchased from commercial vermiculture in the Giza Governorate and maintained in plastic tubs with decomposed organic materials. According to Sucindra Dewi et al.^17^, AcE was extracted with some modifications. Earthworms were washed with water to remove the mucus and soaked in distilled water for 6 h. After being cleaned, worms were cut into small pieces, smoothed, and transferred into a glass beaker containing ethanol (80%). Then, samples were centrifuged for 10 min at 3000 rpm. Finally, the supernatant was collected and evaporated in a water bath and oven for complete dryness.

Extraction of AcCF was conducted by the thermal shock method, according to Dinesh^18^. The earthworms were put in a dry clean petri dish on a hot plate (55–60 °C), and the fluid was collected and lyophilized.

### Characterization of AcE and AcCF composition

#### Gas chromatography–mass spectrometry analysis (GC–MS) analysis

AcE and AcCF were analyzed using Trace GC1310-ISQ mass spectrometer (Thermo Scientific, Austin, TX, USA) with a direct capillary column TG–5MS. The column oven temperature was initially held at 50 °C and then increased by 5 °C /min to 230 °C for 2 min. The final temperature was 290 °C by increasing 30 °C /min and hold for 2 min. The injector and MS transfer line temperatures were kept at 250 °C and 260 °C; Helium was used as a carrier gas at a constant flow rate of 1 ml/min. Diluted samples of AcE and AcCF (1 µl) were injected automatically using Autosampler (AS1300) coupled with GC in the split mode with a solvent delay of 3 min. Electron ionization mass spectra were collected at 70 eV ionization voltages with a scan range of 40–1000 m/z. The ion source temperature was set at 200 °C. Total GC running time was 35 min. The components were identified by comparison of their retention times and mass spectra with those of WILEY 09 and NIST 11 mass spectral databases.

#### Inductive coupled plasma mass spectrometry ICP-MS

AcE and AcCF were digested for 16 h in a mixture containing 15 ml of nitric acid (HNO3, 65%) and 15 ml of perchloric acid (HClO_4_, 70%). After complete evaporation, 5 ml of 10% HCl was added to the samples, and the volume was adjusted to 25 ml with distilled water. Before analysis, these solutions were kept in polyethene tubes at 4 °C. All samples were digested using microwave settings (15-min, steady increase in temperature to 180 °C and 800 W). samples were then analyzed using Inductively Coupled Plasma (6500 Duo, Thermo Scientific, England). 1000 mg/L multi-element certified standard solution (Merck, Germany) was utilized as a stock solution for standardization.

#### Determination of calcium content of AcE and AcCF

Calcium content was determined as one gram of the prepared AcE, and AcCF powder was dissolved in distilled water and then measured according to the manufactured biodiagnostic spectrum kit (Al Obour, Cairo Governorate, Egypt).

In vitro Biological studies of the AcE and AcCF

Determination of the antioxidant activity of AcCF and AcE using the DPPH protocol.

According to Lu et al.^19^, the DPPH protocol was followed. Briefly, 0.1 mM DPPH solution was prepared by dissolving 4 mg of DPPH in 100 ml of methanol. Then, 40 µL of different concentrations of AcE and AcCF (10, 20, 30,40 ,50, 60 mg/mL) was added into different tubes containing 2.96 mL DPPH (0.1 mM) solution. The reaction mixture was incubated in the dark at room temperature for 20 min. Then absorbance (Abs) was read at 517 nm using Clinichem (Biomed Diagnostics, White City, USA).

The % radical scavenging activity of the AcE and AcCF was calculated using the following formula:

$$\% {\text{RSA}} = \frac{Abs control - Abs sample }{{Abs control}} \times 100$$

RSA is a radical scavenging activity.

Abs control is the absorbance of DPPH radical + methanol.

Abs sample is the absorbance of DPPH radical + AcE or AcCF.

Anti-inflammatory activity of AcCF and AcE using Heat-induced hemolysis protocol.

The hemolysis test was performed according to Luna et al.^20^ In brief, erythrocytes were separated from plasma by centrifugation of the whole blood samples at 3000 rpm and washed three times with isosaline of equal volume (0.85%, pH 7.2). Erythrocytes were resuspended in 10% v/v isosaline suspension. As a control, 1 ml of distilled water was mixed with 1 ml of 10% RBCs. Varied concentration of AcCF and AcE (10, 50, and 100 mg/mL) was added to 1 ml of 10% RBCs. After 30 min of incubation of the reaction mixture in a water bath at 56 °C, the tubes were cooled under a running tap. The reaction mixture was centrifuged for 5 min at 2500 rpm, and the supernatants' absorbance was measured at 560 nm. The percentages of hemolysis and protection were calculated according to the following formula:

$${\text{Hemolysis}}\% \, = \frac{Optical density of test sample}{{Optical density of control}} \times 100$$

$${\text{Protection }}\% \, = 100 - \left[ { \frac{Optical density of test sample}{{Optical density of control}} \times 100} \right]$$

### Experimental animals

Adult male CD-1 mice (*Mus musculus*) were used in all experiments. Animals with an average body weight of 38–40 g were bought from the National Research Center (NRC, Cairo, Egypt), housed in polypropylene cages (10 animals/cage) at a temperature of (22–25) °C under 12:12 h day/night cycles, and supplied with a standard laboratory diet and water ad libitum. All the experimental procedures were carried out following international guidelines for the care and use of laboratory animals.

### Ethical approval

Experimental protocols and procedures were approved by Cairo University Institutional Animal Care and Use Committee (CU-IACUC) (Egypt) (CU/I/F/73/20). Animal handling and experimentation complied with the ethical standards established by the Egyptian animal welfare laws and regulations and were performed in line with the Guide for the Care and Use of Laboratory Animals, 8th edition. The authors confirm that the study was performed in accordance with ARRIVE guidelines.

### Acute toxicity test (LD_50_)

LD_50_ of AcCF and AcE was determined according to Chinedu et al.^21^ The mice were starved overnight and then divided into four groups (n = 2), each for AcCF and AcE (2 mice per dose). AcCF and AcE doses of 10, 100, 300, and 600 mg/kg were intraperitoneally injected. The animals were monitored for 1 h after injection, then 10 min every 2 h for the next 24 h. In addition to death, the animals were observed for any changes in behavior such as paw licking, weariness, semi-solid faeces, salivation, writhing, and loss of appetite. LD_50_ of AcE = $\frac{M0+M1}{2}= \frac{300+600}{2}=450$, and AcCF dose was = $\frac{100+300 }{2}=200$, where M0 is the greatest dose that did not cause mortality and M1 is the lowest dose that did cause mortality. AcCF dose is 1/10 of LD_50_ = 20 $mg/kg$, AcE dose is 1/10 of LD_50_ = 45 $mg/kg.$

### Experimental design

Osteoporosis was induced in mice by subcutaneous injection of 1 mg/kg dexamethasone five times per week for 28 days^9^. The control group was injected with 0.9% saline. Mice were randomly separated into four groups (n = 10 per group) and concurrently treated as follows:

Group I Control: Animals were injected intraperitoneally with distilled water every other day for 28 days.

Group II glucocorticoid-induced osteoporosis model (GIOP): Animals were injected intraperitoneally with distilled water every other day for 28 days.

Group III GIOP and AcCF: Animals were injected intraperitoneally with AcCF (20 mg/kg body weight) every other day for 28 days.

Group IV GIOP and AcE: Animals were injected intraperitoneally with AcE (45 mg/kg body weight every other day for 28 days.

At the end of the experiment, animals were anaesthetized by sodium pentobarbital (50 mg/kg). Bone mineral density (BMD) and bone mineral content (BMC) of the femur were analyzed using Dual-energy X-ray absorptiometry (DEXA). Blood samples were collected by retro-orbital method from the eye of the mice, then collected in centrifuge tubes without anticoagulant, then centrifuged at 3000 rpm for 10 min, and the collected sera were stored at -80 °C until use. Left femora were excised, muscle and connective tissues were removed, weighed, and homogenized in 50 mM phosphate buffers (pH 7.4). The homogenate was centrifuged for 15 min at 4 °C at 860 xg, and the supernatant was used for biochemical analysis.

### Biochemical analysis

Calcium and phosphorus contents were measured in the homogenate of the femur bone, bone alkaline phosphatase (BALP), serum alkaline phosphatase (ALP), bone acidic phosphatase (BACP) and serum acidic phosphatase (ACP) were determined according to manufacture biodiagnostic kits from the spectrum (Al Obour, Cairo Governorate, Egypt). Parathyroid hormone and calcitonin were measured using an enzyme‐linked immunosorbent assay (ELISA) kit from Bioassay technology laboratory (Shanghai, China). Bone supernatants were used for the determination of malondialdehyde (MDA), nitric oxide (NO), glutathione reduced (GSH) and superoxide dismutase (SOD) according to manufacture kits from Bio Diagnostic (El Omraniya, Giza Governorate, Egypt).

### Histopathological investigation

#### Bone histomorphometry

The femurs were fixed for two days in 10% neutral buffered formalin, then decalcified in 10% EDTA (pH 7.4) for two weeks before being embedded in paraffin. Along the coronal plane, the samples were sliced into 5-µm thick slices; sections were stained with hematoxylin and eosin (H&E) to examine histological parameters of trabecular and cortical bones using a light microscope (OPTRCH, Germany)^22^.

#### Alcian blue staining of Glycosaminoglycans (GAGs)

Alcian blue staining is a semiquantitative method to measure GAG contents^23^. Bone sections were incubated for 3 min in 3% acetic acid, then in 1% Alcian blue 8GX (Sigma-Aldrich, St. Louis, MO, USA), pH 2.5 for 40 min at room temperature, followed by destaining in water and counterstained with nuclear fast red.

#### Real-time polymerase chain reaction (RT-PCR) analysis

Tibias were cleaned of muscle and connective tissue, snap-frozen in liquid nitrogen, and stored at -80 °C. Frozen tibias were crushed in liquid nitrogen with a pestle and mortar. According to the manufacturer's instructions, total RNA was extracted using a GeneJET RNA purification kit (Thermo Scientific™, Massachusetts, USA). And first-strand complementary DNA (cDNA) was reverse-transcribed from total RNA (1 μg) using H Minus cDNA Synthesis Master Mix Kit (Thermo Scientific™, Massachusetts, USA). Changes in gene expression levels were quantified using Simply Green qPCR Master Mix, low ROX and specific primers. Using Step One Plus Real-Time PCR System (Applied Biosystems, CA, USA), the relative expression of each target gene was measured using 2 − ΔΔCt. Data were normalized to the housekeeping gene beta-actin. PCR primers (forward and reverse, respectively) were as follows:

*Actb* (5’-GGCTGTATTCCCCTCCATCG-3’ and 5’-CCAGTTGGTAACAATGCCATGT-3’), *RANKL* (5'-GTGAAGACACACTACCTGACTCC-3' and 5'-GCCACATCCAACCATGAGCCTT-3'), *OPG* (5'-CGGAAACAGAGAAGCCACGCAA-3' and 5'-CTGTCCACCAAAACACTCAGCC-3'), *Rank* (5'-CCAGGAGAGGCATTATGAGCA-3' and 5'-ACTGTCGGAGGTAGGAGTGC-3'), *TRAP* (5'-ACCAGCAAGGATTGCGAGGCAT-3' and 5'-GGATGACAGACGGTATCAGTGG-3'), *Sdc-1* (5'-GACAGAGGTAAAAGCAGTCTCG-3' and 5'-CTTTGTCACGGCAGACACCTT-3'), *Mmp2* (5’-CAAGGATGGACTCCTGGCACAT-3’ and (5’-TACTCGCCATCAGCGTTCCCAT-3’), *Mmp9*(5’-GCTGACTACGATAAGGACGGCA-3’ and 5’-TAGTGGTGCAGGCAGAGTAGGA-3’).

### Statistical analysis

Statistical analysis was conducted using GraphPad Prism 8.0 (GraphPad Software, USA) and SPSS for Windows (version 15.0). All data are expressed as mean ± standard deviation (SD). The comparisons within groups were tested using one-way analysis of variance (ANOVA) with the Duncan post hoc test, and *P *< 0.05 was considered statistically significant was used for the statistical analysis.

## Results

### Characterization of AcCF and AcE

#### *Gas chromatography-mass spectrometry (GC–MS) analysis*

The dominant compounds found in AcE and AcCF are fatty acids such as hexadecenoic acid, octadecanoic acid, pentadecanoic acid, and dodecanoic acid, which have potential biological activity and thus acts as an antioxidant, antibacterial, and anti-inflammatory^24,25^. The compounds are listed in supplemental tables 1 and 2.

#### Inductive coupled plasma mass spectrometry (ICP-MS)

ICP analysis of AcE and AcCF has revealed safe limits of heavy metals^26^. Metal concentration (ppm) of AcCF and AcE, respectively. Zn: (0.0725 and 0.67), Cu: (Nil and 191.13), Cd: (0.01619 and Nil), Pb and Ni: nil in both samples, and Cr: (0.275 and 47.05).

#### *Determination of calcium content in AcE and AcCF*

The present study revealed that the calcium contents of AcE and AcCF were 0.00082 mg/g and 0.0228 mg/g, respectively.

#### In vitro biological activities of AcCF and AcE

**Figure 1:**
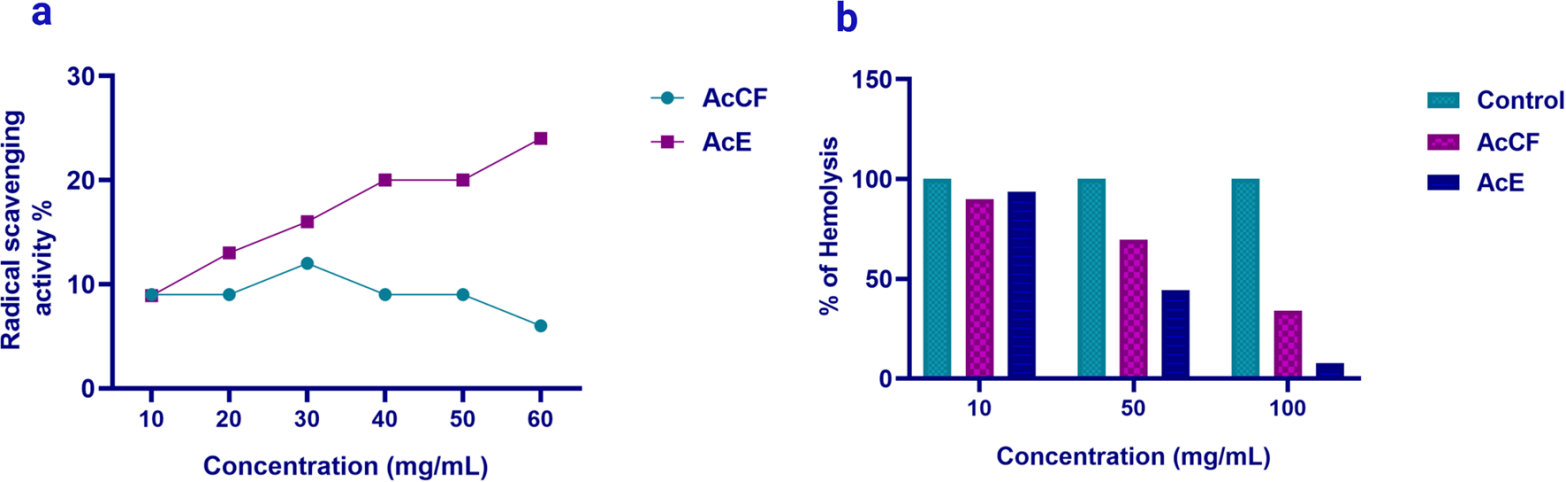
In vitro biological activities of AcCF and AcE. (**a)** Antioxidant activity assayed by radical scavenging activity of DPPH. **(b)** Anti-inflammatory activity using heat-induced hemolysis.

Both AcCF and AcE exerted in vitro antioxidant and anti-inflammatory activities. However, AcE was more potent in scavenging free radicals than AcCF (Fig. 1a). Furthermore, AcE reduced hemolysis compared to AcCF in a concentration-dependent manner (Fig. 1b).

#### AcCF and AcE administration significantly enhances BMD and BMC in GIOP mice

**Figure 2:**
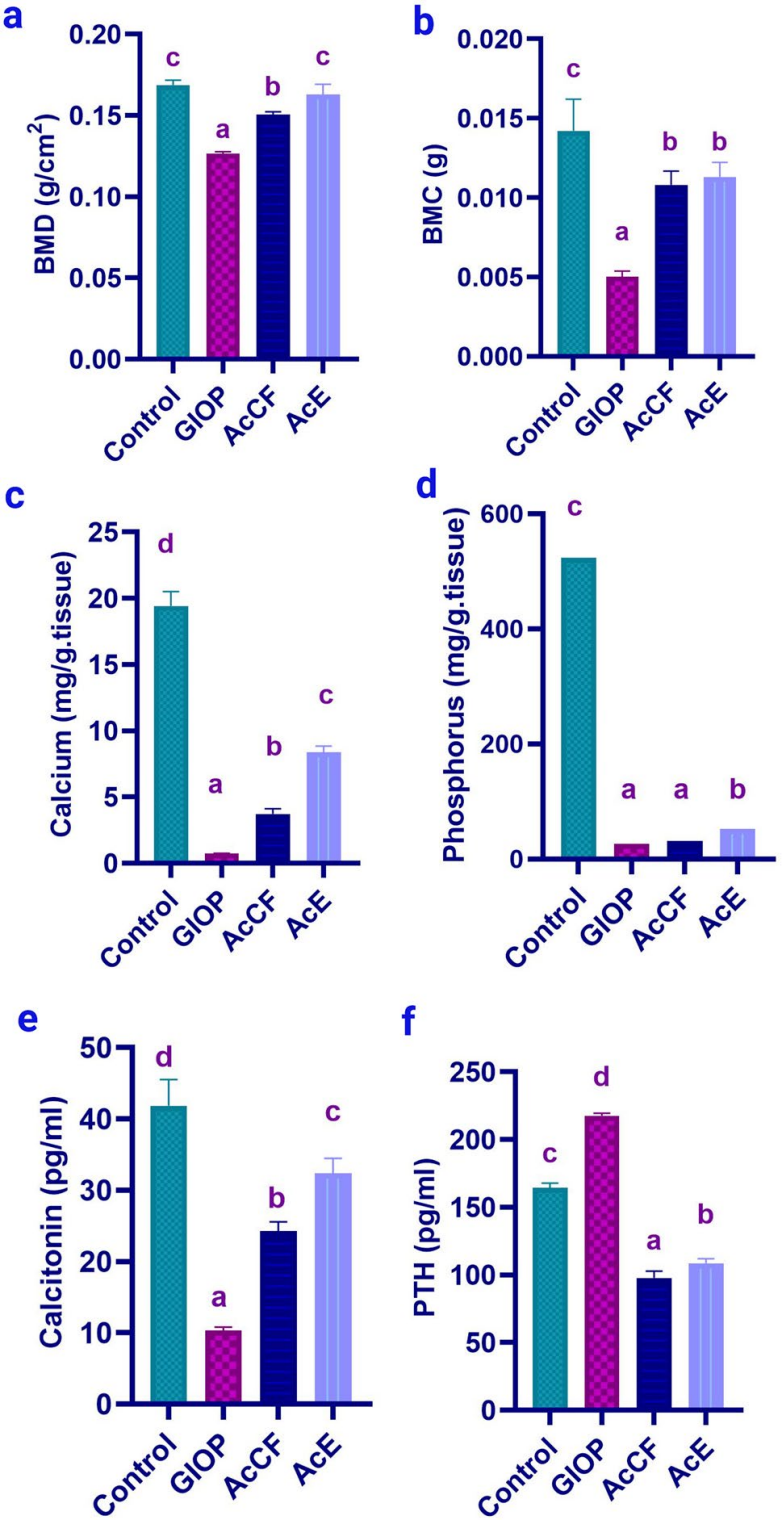
(**a**) Bone mineral density (BMD) assessed by DEXA, (**b)** Bone mineral content (BMC) assessed by DEXA, (**c**) Quantitative analysis of calcium in bone homogenate, (**d**) Quantitative analysis of phosphorus in bone homogenate. **e** Serum levels of calcitonin assessed by ELISA. (**f**) Serum levels of parathyroid hormone (PTH) assessed by ELISA. Data are expressed as mean ± SD (n = 6 per group). Each value not sharing a common letter superscript is significantly different (*P *< 0.05), where **(a)** is the lowest value and (**d)** is the highest value.

Firstly, Dual-energy X-ray absorptiometry (DEXA) analysis for the femur verified the success of our GIOP model mice, indicating a significant reduction (*P *< 0.05) of BMD and BMC in the GIOP group compared to the control group. Importantly BMD and BMC were significantly augmented upon treatment with AcCF and AcE (Fig. 2a,b, all *P *< 0.05). of note, BMD was markedly restored in the GIOP group treated with AcE to the basal level of normal controls.

#### Homeostasis of calcium and phosphorus content in the bone homogenate after administration of AcCF and AcE

In terms of bone mass, calcium and phosphorus play primary roles^27^ so quantitative analysis of calcium and phosphorus in bone homogenate revealed a significant reduction (*P *< 0.05) in the GIOP compared to the control group (Fig. 2c,d). Administration of AcCF and AcE showed a significant increase in calcium (*P *< 0.05) compared to the GIOP group; meanwhile, the AcE group only showed a significant rise in phosphorus (*P *< 0.05) compared to the GIOP group, which did not happen in the AcCF group.

#### AcE and AcCF administration retain calcitonin and parathyroid hormone (PTH) to their normal levels in the GIOP mice

Calcitonin and parathyroid are the main hormones for bone formation and resorption^28^. ELISA analysis of PTH and calcitonin shows significance (Fig. 2e,f, all *P *< 0.05) of high PTH level of the GIOP group compared to the control group, while calcitonin significantly decreased in GIOP mice compared with the control group. Interestingly, administration of AcCF and AcE restored significantly (*P *< 0.05) the serum calcitonin and PTH levels near their standard value.

#### Effect of AcE and AcCF on histological morphology of the femur bone

**Figure 3:**
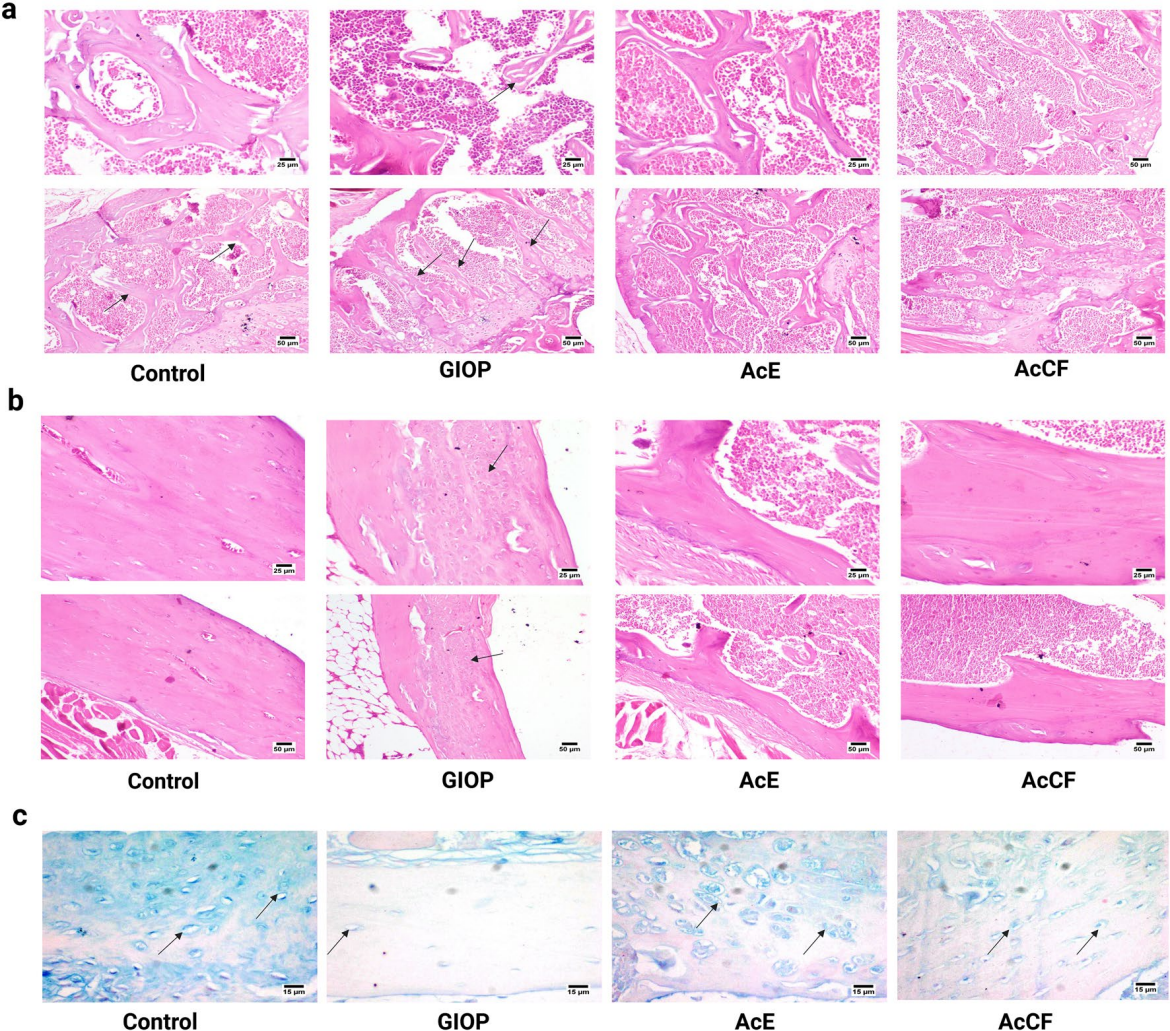
Histological analysis of the trabeculae and cortical bones in the femur of control, GIOP, AcE and AcCF. (**a**) Photomicrograph of bone trabeculae (arrows) (H&E). Control group showing normal trabeculae. GIOP group showing thin bone trabeculae with defective mineralization. AcE group showing normal bone trabeculae. AcCF group showing mild thinning in the bone trabeculae. (**b**) Photomicrograph of compact bone (H&E). The Control group showed normal compact bone. GIOP group showing defective mineralization of compact bone (arrow). AcE group showing normal compact bone. AcCF shows normal compact bone. (**c**) Photomicrograph of bone stained with Alcian blue. Control group showing normal GAGs deposition. GIOP group showing marked reduction in GAGs deposition. AcE group showing increased GAGs deposition. AcCF- group showing a mild increase in GAGs deposition. Notably, AcE shows better GAGs deposition. Arrow: GAGs deposition.

The thickness of trabeculae and cortical bone has been investigated by H&E staining (Fig. 3a,b). Microscopic examination of bone sections from the control group revealed normal trabecular bone that appeared thick and continued; the compact bone was normal with normal calcification. The GIOP group's bone trabeculae were short and incomplete, resulting in wide marrow spaces. The bone trabeculae showed defective mineralization. Compact bone showed defective calcification with the presence of osteoid tissue. AcE group showed normal bone trabeculae, and the shaft was also histologically normal. Concerning the AcCF group, the examined sections showed a mild decrease in the bony trabeculae's thickness at the bone's head with a compact shaft bone.

#### AcE and AcCF regenerate the organic compartment by enhancement of glycosaminoglycans (GAGs)

The examined bone sections stained with Alcian blue revealed normal GAGs deposition in the control group (Fig. 3c). Although a marked reduction of GAGs was noticed in the GIOP group, AcE, and AcCF treatment augmented GAGs deposition. Notably, GAGs staining was higher in the AcE-treated group than in the AcCF-treated group.

#### Effect of AcE and AcCF administration on activity of osteoblast/osteoclast-related markers

**Figure 4:**
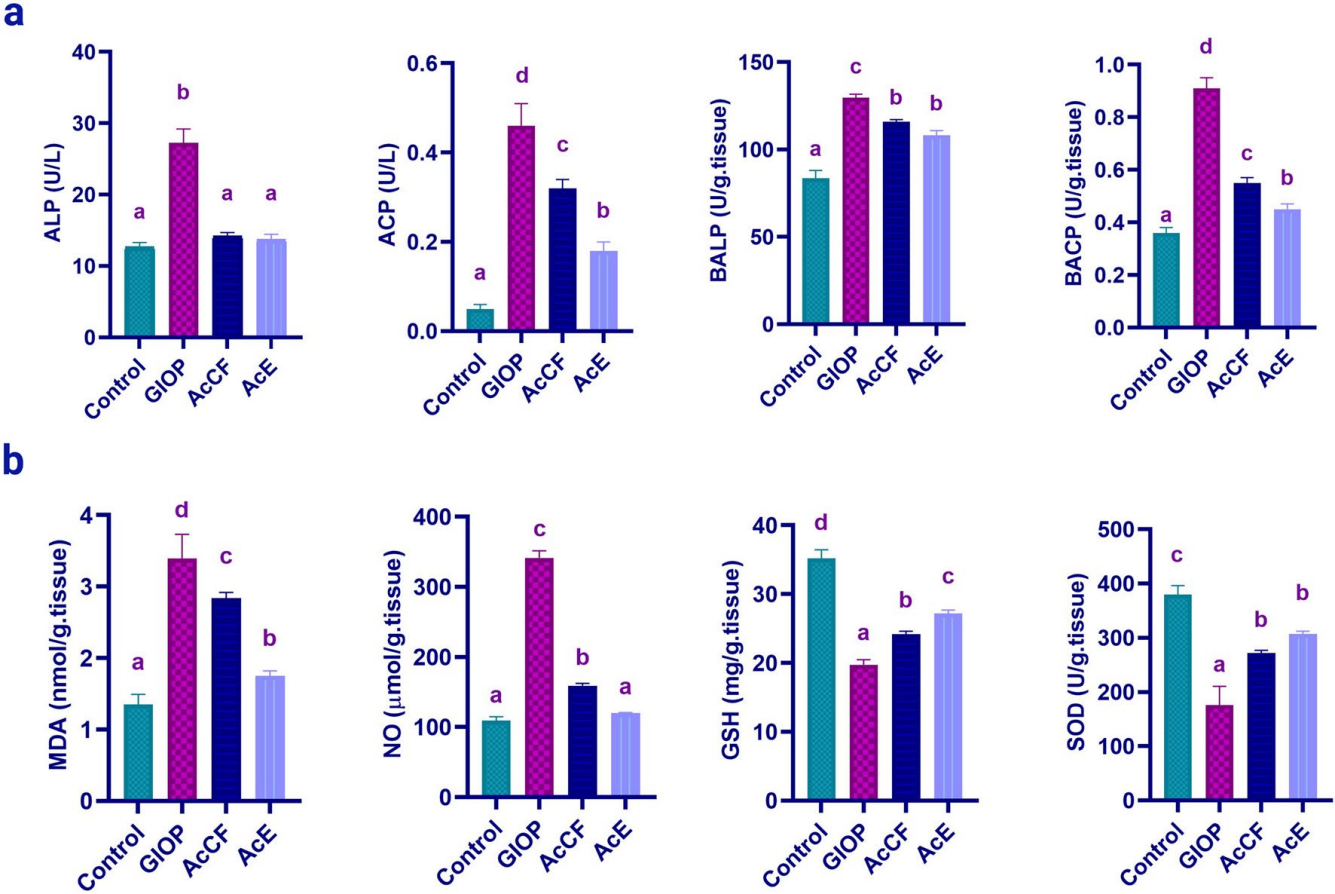
(**a**) Effect of AcCF and AcE on the activity of osteoclast/osteoblast related markers; Serum alkaline phosphatase (ALP), serum acidic phosphatase (ACP), bone alkaline phosphatase (BALP), and bone acidic phosphatase (BACP). (**b**) Effect of AcE and AcCF on oxidative stress markers; malondialdehyde (MDA), nitric oxide (NO), glutathione reduced (GSH), and superoxide dismutase (SOD). Data are expressed as mean ± SD (n = 6 per group), and each value not sharing a common letter superscript is significantly different (*P *< 0.05), where (**a**) is the lowest value and (**d**) is the highest value.

Metabolites evaluate the effect of AcE and AcCF on osteoblast and osteoclast activity, showed in Fig. 4a.

The activity of serum alkaline phosphatase (ALP) showed a significant increase in the GIOP group (*P *< 0.05) compared to the control group. AcCF and AcE administration showed a significant decrease (*P *< 0.05) in ALP activity compared to the GIOP group.

Serum acidic phosphatase (ACP) has been significantly increased in the GIOP group (*P *< 0.05) compared to the control group; meanwhile, AcCF and AcE groups induced a significant reduction of ACP compared to the GIOP group (*P *< 0.05), there is a significant decreasing result of AcE group compared to AcCF group.

Bone alkaline phosphatase (BALP) increases significantly (*P *< 0.05) in the GIOP group compared to the control group. AcCF and AcE groups showed a significant decrease in BALP (*P *< 0.05) compared to the GIOP group.

Bone Acidic phosphatase (BACP) increased significantly in the GIOP group (*P *< 0.05) compared to the control group, and AcCF and AcE showed a significant decrease in acidic phosphatase activity (*P *< 0.05) compared to the GIOP group.

#### Administration of AcE and AcCF suppresses oxidative stress in GIOP mice

Since the progression of GIOP is inextricably linked to oxidative stress, we managed to evaluate oxidative stress markers^16^ (Fig. 4b). GIOP group showed a significant increase in the MDA (*P *< 0.05) compared to the control group. Interestingly, AcCF and AcE showed a significant decrease (*P *< 0.05) in MDA compared to the GIOP group.

Consequently, NO has significantly increased in the GIOP group (*P *< 0.05) compared to the control group. The verses happened in the groups; AcCF and AcE showed a significant decrease (*P *< 0.05) compared to the GIOP group.

Antioxidants, GSH, and SOD have decreased significantly (*P *< 0.05) in the GIOP group compared to the control group. Meanwhile, the AcCF and AcE groups increased GSH, and SOD significantly (*P *< 0.05) compared to the GIOP group.

#### AcE and AcCF administration downregulate the expression of RANK and Mmp9 in GIOP mice

**Figure 5:**
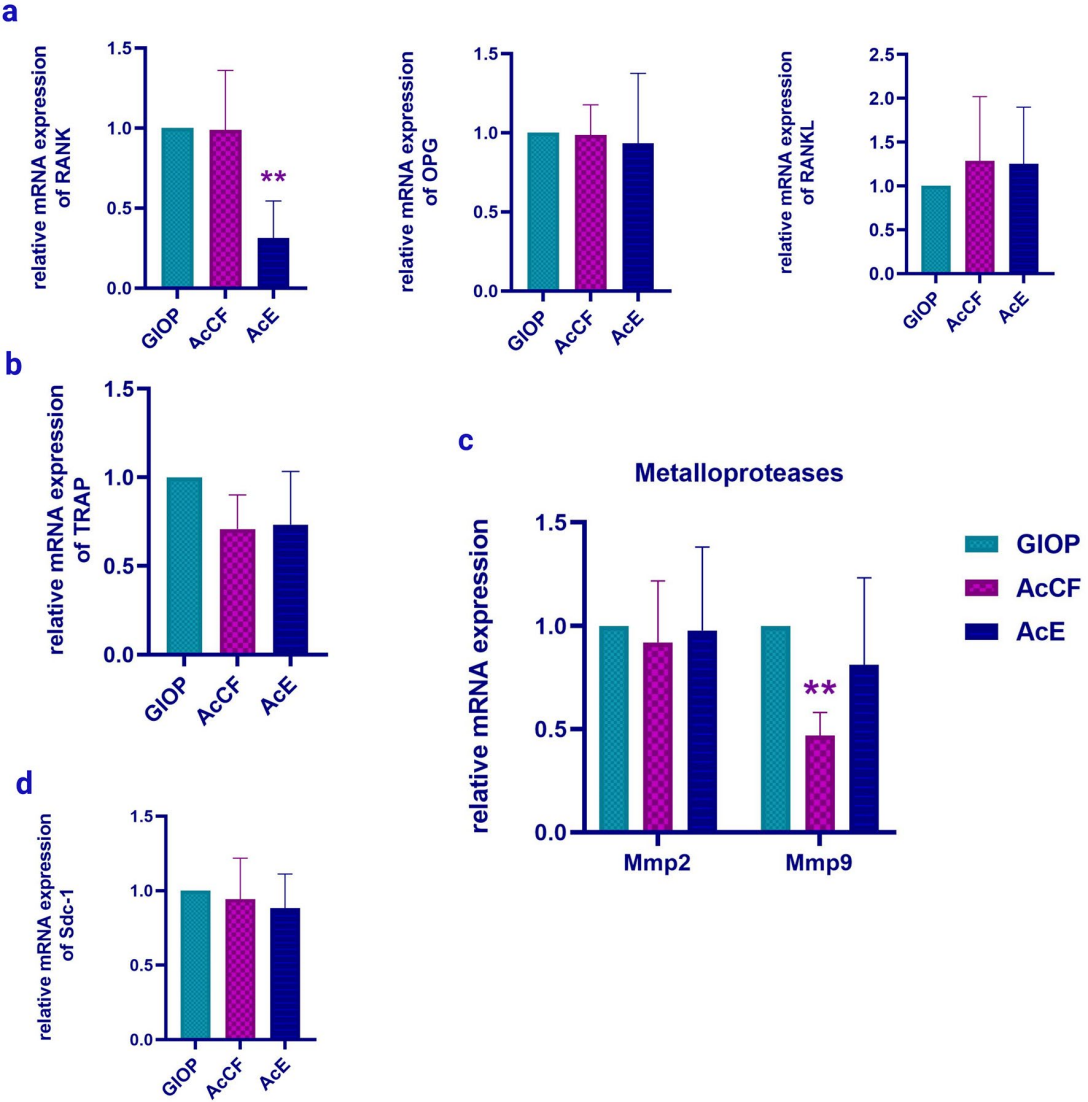
Quantitative real-time RT- PCR shows the effect of AcCF and AcE on the expression levels of (**a**) RANK, OPG and RANKL, (**b**)Tartrate acidic phosphatase (TRAP), (**c**) Matrix metalloproteases; Mmp2 and Mmp9 d Syndecan-1 (Sdc1). Data are expressed as mean ± SD (n ≥ 3), *P < 0.05, **P < 0.01.

It is well known that OPG, RANKL, and RANK constitute a system that regulates bone remodelling^29^. In the AcE group, the RANK expression has significantly decreased (*P *< 0.01) compared to the GIOP group (Fig. 5a). Although AcE and AcCF have a considerable impact on some osteoclastic differentiation markers, TRAP gene expression has not significantly affected by AcE and AcCF (Fig. 5b). However, Mmp9 has decreased significantly (*P *< 0.05) in the AcCF group (Fig. 5c), there is no significant results regarding Sdc1 and Mmp2 (Fig. 5d).

## Discussion

Bone loss in GIOP mice was prevented by both AcCF and AcE, and to the best of our knowledge, this is the first study showing the effects of AcCF and AcE on GIOP. AcE, in particular, had a more noticeable impact than AcCF did on the vast majority of measured variables.

The most relevant characteristic of GIOP is a bone fracture due to a decrease in BMD and BMC^30^. DEXA is the most commonly used method for measuring BMD of small animals used in the study of a wide spectrum of metabolic bone disease studies^31^. It is a simple, fast, low-radiation, and cost-effective alternative to micro-computed tomography (micro-CT) used for quantitative analysis of changes in the bone in living subjects, which enables researchers to obtain BMD values at different time points in longitudinal studies than micro-CT^32–34^.It is known that calcium and phosphorus, the main components of hydroxyapatite crystals, affect BMD directly and indirectly^27^. GIOP increases renal calcium loss and decreases gastrointestinal absorption of calcium^35^. The present study revealed a significant decrease in BMD, BMC, calcium, and phosphorus in the GIOP group compared to the control group, leading to hypomineralization of the bone matrix. These findings were corroborated by histological investigation of the femur, which revealed weakening in the GIOP group's trabeculae thickness, which was preserved by AcE and AcCF injection. AcE and AcCF increased calcium and phosphorus, increasing BMD and BMC. Quantitative analysis of AcE and AcCF composition uncovered high calcium and zinc concentrations. It was reported that calcium plays a role in the chondroitin sulfate calcium complex that evolves in chondrocyte growth leading to regeneration of bone matrix^36^, and zinc increase matrix calcification^37^. The finding is consistent with the recent study showing increased differentiation and maintenance of osteoblasts by zinc and calcium.

Changes in enzymes associated with bone metabolism were also covered in this investigation. ALP and BALP are frequently used to indicate bone metabolic activity and track drug response during treatment^3^. It was reported that ALP and BALP increase in GIOP because dexamethasone causes osteoblast apoptosis, spreading osteoblast content that raises the enzymes in bone and serum^38^, which explains the elevated serum ALP and BALP activity in dexamethasone-treated mice of this study. ACP is the enzyme that evolved in bone matrix degradation correlated with bone acidic phosphatase (BACP), which is secreted by osteoclasts^39^. ACP and BACP also increased in the GIOP group compared to the control group because of bone resorption^40^. Treatment with AcE and AcCF decreases ALP, BALP, ACP, and BACP to the normal level. AcE has been shown to diminish bone resorption and suppress osteoblast apoptosis, hence reducing enzyme activity^41^.

PTH stimulates calcium release in an indirect process through osteoclasts, ultimately leading to bone resorption. Unlike PTH, calcitonin inhibits calcium withdrawal from the bone and decreases osteoclasts' lytic effect^42^. Surprisingly, PTH was significantly increased in the GIOP group compared to the control group, while calcitonin level has reduced in the GIOP group than in the control group. Hyperparathyroidism in GIOP is attributed to calcium deficiency^35^. Intriguingly, treatment with AcE and AcCF optimizes PTH and calcitonin levels, which may be a consequence of increased mineral incorporation and osteoblast synthesis^41^.

Several lines of evidence correlate GIOP with increased reactive oxygen species (ROS) and antioxidant depletion^35^. A significant increase in MDA and NO, whereas a decrease in antioxidants such as GSH and SOD in GIOP was observed compared to the control group. It was reported that GCs administration induces osteoporosis by enhancing oxidative stress^43^. AcE and AcCF-treated groups showed a decrease in MDA and NO but an increase in GSH and SOD. The antioxidant effect of AcE and AcCF is probably caused by their high content of fatty acids, where hexadecenoic acid, octadecenoic acid, decanoic acid, and other compounds have revealed antioxidant properties^24,25^.

In terms of osteoclastogenesis and bone resorption, the RANK/RANKL pathway is primarily responsible for the mechanism of action^44^. As a result, antiresorptive efficacy is acquired by the downregulation of RANK^6^. During bone resorption, mature osteoclasts require Mmp9, a degradative enzyme of the extracellular matrix^45^; thus, Mmp9 has been recognized to be one of the osteoclast-specific markers^46^. Intriguingly, treatment with AcCF have significantly downregulated the expression of Mmp9 , the downstream target of NF-kB activation mediated by RANK/RANKL signaling^47^, and AcE have significantly downregulated the expression of RANK compared to the GIOP group, indicating a decreased lytic effect of osteoclasts^44,48^.

Damage of GIOP to the extracellular matrix (ECM) has gone further to the organic compartment. In bone, the extracellular matrix composition varies. It is composed of up to 20–40% of organic components^49^. The predominant constituent of the organic ECM is significant structural proteins such as collagen complemented with proteoglycans (PGs), glycosaminoglycans (GAGs), and other proteins^50^. GAGs are highly negatively charged polysaccharides that preserve the extracellular matrix's structural integrity and viscosity. Our histological investigation has shown depletion of GAGs deposition in the GIOP group compared to the control group. GIOP-induced inflammatory secretions are the reason for GAGs reduction. Remarkably, treatment with AcE and AcCF has increased the GAGs content; however, AcE showed better retaining of GAGs than AcCF, indicating increased bone toughness. Enrichment of earthworms with acidic polysaccharides may attribute to enhanced GAGs depositions^51^.

## Conclusion

**Figure 6:**
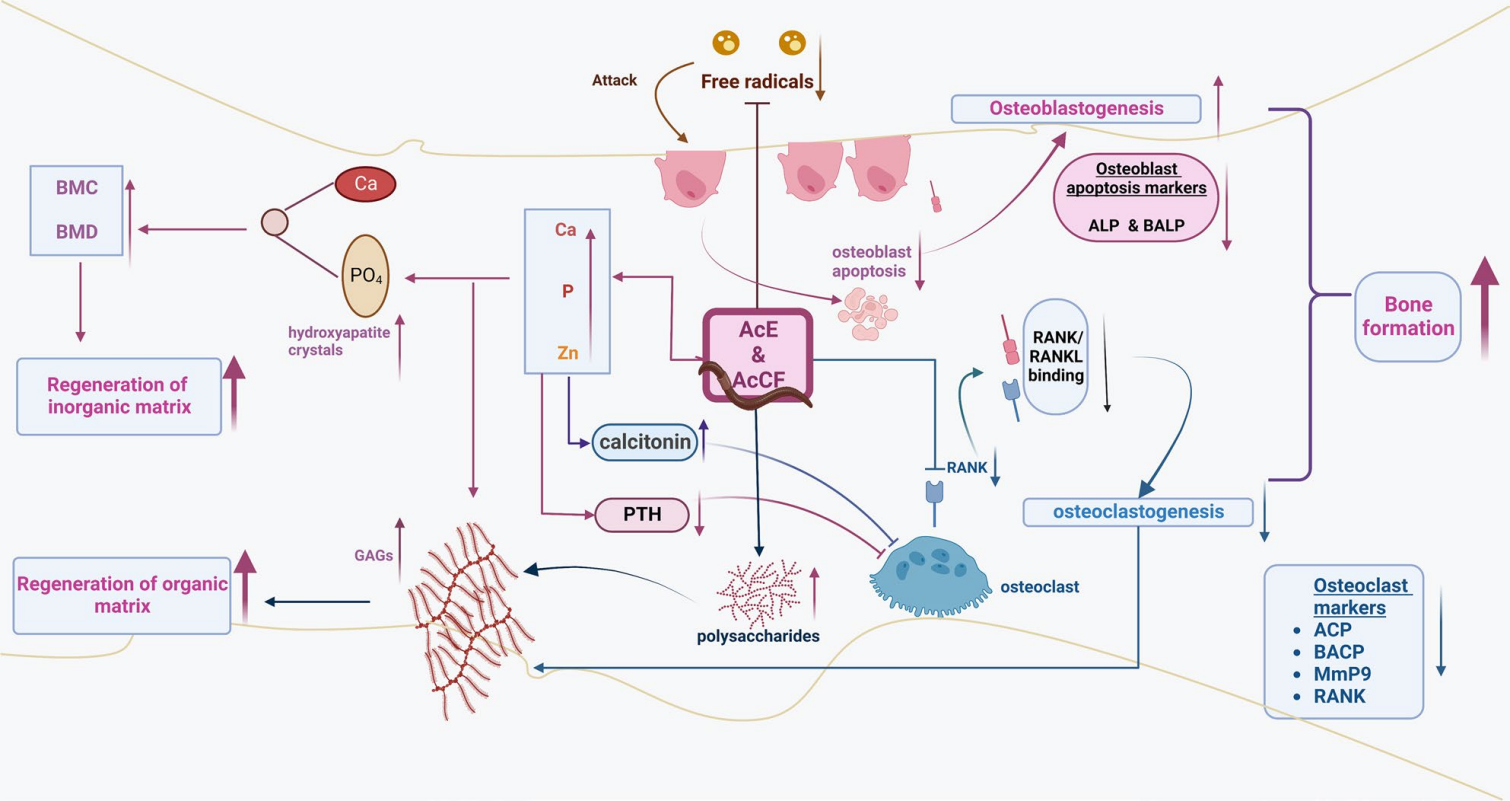
Schematic diagram showing the chemo-preventive potency of AcCF and AcE on bone compartments via multiple pathways against GIOP; (1) bone cells, (2) inorganic matrix, and 3) organic matrix. Through antioxidant effect, AcE and AcCF treatment inhibit osteoblast apoptosis. Osteoclastogenesis has been decreased by RANK downregulation, which causes the RANK/RANKL pathway to malfunction. Calcium, phosphorus, and zinc content in AcE and AcCF promote the renewal of the inorganic matrix by increasing BMD and BMC and aiding in hormone homeostasis (PTH and calcitonin). Calcium and zinc increased GAGs formation by forming chondroitin sulfate calcium complex. Polysaccharide content in AcE and AcCF has increased GAGs content, regenerating the organic bone matrix.

This study has revealed a new mechanism regarding the action of AcE and AcCF on GIOP (Fig. 6). Bone regeneration occurred by three main processes: 1- Bone cell balance, (a) The antioxidant effect of AcE and AcCF has decreased osteoblast apoptosis, leading to decreasing apoptosis markers; ALP and BALP. (b) osteoclast formation has decreased due to down-regulation of RANK, leading to malfunctioning of the RANKL/RANK pathway. (d) AcE and AcCF have decreased osteoclast markers which are ACP, BACP, and Mmp9. So, osteoblast formation and osteoclast inhibition increased bone formation; 2-Regeneration of the in-organic matrix, (a) Calcium and phosphorus content in AcE and AcCF increased BMD and BMC, thus forming hydroxyapatite crystals; (b) Because of calcium and phosphorus homeostasis PTH decreased to a normal level; while, calcitonin increased to the normal level; (c) Osteoclast lytic activity is diminished after decreasing PTH and increasing calcitonin; 3- Regeneration of GAGs in the organic bone matrix (osteoid) may be attributed to: a) polysaccharide content in AcE and AcCF that help in GAGs formation, b) AcE and AcCF enhance the formation of chondroitin sulfate calcium complex in the presence of calcium and zinc, increasing GAGs content; c) downregulation of Mmp9, decreases degradation of the bone matrix. AcE and AcCF lead to the balance of bone cells and repair bones' organic and inorganic compartments. Therefore, AcE and AcCF may emerge as promising prospects for developing a potential natural chemo-preventive product therapy for GIOP. Further research is needed to define the specific component(s) (amino acid, fatty acid, or polysaccharide) of AcE and AcCF that may ameliorate GIOP.

## Supplementary Information

Supplementary Tables.

## Data Availability

All data generated or analyzed during this study are included in this published article.
