# Supplementary material for: Allolobophora caliginosa coelomic fluid and extract alleviate glucocorticoid-induced osteoporosis in mice by suppressing oxidative stress and regulating osteoblastic/osteoclastic-related markers
Source: Sci Rep. 2023 Feb 6;13:2090. doi: 10.1038/s41598-023-29070-5 (PMC9902447; doi:10.1038/s41598-023-29070-5)
Supplement: Supplementary file 1 — Supplementary Tables. [file 41598_2023_29070_MOESM1_ESM.pdf]

“Allolobophora caliginosa coelomic fluid and extract alleviate glucocorticoid-induced osteoporosis in mice by suppressing oxidative stress and regulating osteoblastic/osteoclastic-related markers”

Authored by:

1. Ms. Marwa Ahmed Abdelfattah*

Demonstrator
Zoology Department - Faculty of Science - Cairo University
12613 Giza, Egypt.
Email: maabdelfattah@sci.cu.edu.eg
maabdelfattah@cu.edu.eg
cell phone: +201000955956
Fax. +2-02-357-275-56

2. Dr. Ayman Saber Mohamed

Associate professor of Physiology
Zoology Department - Faculty of Science - Cairo University
12613, Giza, Egypt.
Email: ayman81125@hotmail.com
ayman81125@cu.edu.eg
cell phone: +201275350954

3. Dr. Sherif Abdelaziz Ibrahim

Associate professor of cell and cancer biology
Zoology Department - Faculty of Science - Cairo University
12613 Giza, Egypt.
Email: isherif@cu.edu.eg
isherif@sci.cu.edu.eg
cell phone: +201004858517

4. Prof. Dr. Sohair R. Fahmy

Professor of physiology
Zoology Department - Faculty of Science - Cairo University
12613 Giza, Egypt.
Email: sohairfahmy@gmail.com
sohair@sci.cu.edu.eg

Table 1. Chemical components identified in the *Allolobophora calignosa* coelomic fluid by GC-MS analysis.

Compound name	Retention time	Peak area	Molecular weight	Molecular formula
DL-2-Amino-3-phosphonopropionic Acid	6.97	1.1E+08	169	$C_3H_8NO_5P$
2-Pyridinecarboxaldehyde	10.9	1.5E+08	107	C_6H_5NO
Hirsutene	13.69	2.4E+09	204	$C_{15}H_{24}$
(1-Bromo-ethanesulfinyl)-ethane	14	2.3E+08	184	C_4H_9BrOS
1-Propene, 3,3'-oxybis [2-chloro-	14	2.3E+08	166	$C_6H_8C_{l2}O$
Pentadecanoic acid	26.87	1.9E+09	270	$C_{17}H_{34}O_2$
13-Heptadecyn-1-ol	30	3.9E+08	252	$C_{17}H_{32}O$
2-(9,12-octadecadienoyl)-, (Z,Z)-Ethanol	30	3.9E+08	310	$C_{20}H_{38}O_2$
10-Octadecenoic acid, methyl ester	30.12	2E+09	296	$C_{19}H_{36}O_2$
Methyl 9,10-dideutero octadecanoate	30.12	2E+09	300	$C_{19}H_{36}D_2O_2$
Methyl stearate	30.6	1E+09	298	$C_{19}H_{38}O_2$
Hexadecadienoic acid, methyl ester	31.72	2.1E+08	270	$C_{17}H_{34}O_2$
[1,1'-Bicyclopropyl]-2-octanoic acid, 2'-hexyl-, methyl ester	31.72	2.1E+08	322	$C_{21}H_{38}O_2$
9,12-Octadecadienoyl chloride, (Z,Z)-	31.72	2.1E+08	298	$C_{18}H_{31}C_{lO}$
5,8,11,14-Eicosatetraenoicacid, methyl ester, (all-Z)-	32.71	2.2E+08	302	$C_{20}H_{30}O_2$
6,9,12- Octadecatrienoic acid, methyl ester	33.69	2.2E+08	312	$C_{19}H_{36}O_3$

Table 2. Chemical components identified in the *Allolobophora calignosa* extract by GC-MS analysis.

Compound name	Retention time	Peak area	Molecular weight	Chemical formula
Dichloromethyl propyl sulfone	5.1	300824933	176	$C_3H_6Cl_2O_2S$
2H-Inden-2-one, octahydro-3a-phenyl-, trans-	5.1	300824933	229	$C_{15}H_{19}NO$
2H-Pyrrole, 2,2-dimethyl-3,5-diphenyl-, 1-oxide	5.1	300824933	263	$C_{18}H_{17}NO$
Spiro[3H-indole-3,2'-pyrrolidin]-2(1H)	5.1	300824933	253	$C_{17}H_{19}NO$
Methyl 2-oxo-7à,10Aá-dimethyl-8á-M	5.1	300824933		
ethoxy-4à-(1-methylethyl4,6,6Aà,7,8,9,10,10A-octahydro-2H-naptho[2,1-C] pyran-7á-carpoxylate	5.1	300824932.89	362	$C_{21}H_{30}O_5$
DL-2-Amino-3-phosphonopropionicacid	9.06	503949458	169	C3H8NO5P
Hexadecanoic acid, methyl ester	26.82	837357023	270	$C_{17}H_{34}O_2$
Pentadecanoic acid, 14-methyl-, methyl ester	26.82	837357023	270	$C_{17}H_{34}O_2$
Cyclopentane tridecanoic acid, methyl ester	26.82	837357023	296	$C_{19}H_{36}O_2$
Hexadecanoic acid	28.98	4174193859.97	256	$C_{16}H_{32}O_2$
Linoleic acid ethyl ester	29.98	662617805	308	$C_{20}H_{36}O_2$
9,12-Octadecadienoic acid, ethyl ester -	29.98	662617805		
Cyclopropane octanoic acid, 2-[[2-[(2-ethylcyclopropyl) methyl] cyclopropyl] methyl]-, methyl ester	29.98	662617805	334	$C_{22}H_{38}O_2$
10-Octadecenoic acid methyl ester	30.11	1.722E+09	296	$C_{19}H_{36}O_2$
Methyl 9,10-dideutero octadecanoate	30.11	1.722E+09	300	$C_{19}H_{36}D_2O_2$
Hexadecanoic acid, 2,3-dihydroxypropyl ester	32.48	1.897E+09	330	C19H38O4
Hi-oleic safflower oil	33.33	380105196	450	$C_{21}H_{22}O_{11}$
9-Octadecenoic acid, 12-hydroxy-, methyl ester, [R-(Z)]-	33.74	2.915E+09	312	$C_{19}H_{36}O_3$
